# 7^th^ Brazilian Guideline of Arterial Hypertension: Chapter 14 - Hypertensive Crisis

**DOI:** 10.5935/abc.20160164

**Published:** 2016-09

**Authors:** MVB Malachias, ECD Barbosa, JFV Martim, GBA Rosito, JY Toledo, O Passarelli Júnior

## Definition

The terms HU and HE were proposed as an operational classification of HC in 1993 by the V *Joint National Committee on Detection Evaluation and Treatment of High Blood Pressure.*^1^ The HUs are symptomatic clinical situations in which there is significant BP elevation (arbitrarily defined as DBP ≥ 120 mm Hg) without acute and progressive TOD.^2,3^ The HEs are symptomatic clinical situations in which there is significant BP elevation (arbitrarily defined as DBP ≥ 120 mm Hg) with acute and progressive TOD.^2,3^

Patients complaining from headache, atypical chest pain, dyspnea, acute psychological stress, and panic syndrome associated with high BP levels characterize neither HU nor HE, but rather a pseudo hypertensive crisis. Treatment comprises the optimization of antihypertensive drugs and raising awareness about treatment adherence.

### Classification

Chart 1 shows the classification of HE, and Chart 2 differentiates HU from HE regarding diagnosis, prognosis and management.

**Chart 1:** Classification of hypertensive emergencies

HYPERTENSIVE EMERGENCIES
Cerebrovascular - Hypertensive encephalopathy - Intracerebral hemorrhage - Subarachnoid hemorrhage - Ischemic stroke Cardiocirculatory - Acute aortic dissection - APE with left ventricular failure - AMI - Unstable anginaRenal - Rapidly progressive AKI
Severe adrenergic crises Crisis of PHEO Excessive dose of illicit drugs (cocaine, crack, LSD)
Pregnancy hypertension Eclampsia Severe preeclampsia “HELLP” syndrome Severe hypertension at the end of pregnancy

APE: acute pulmonary edema; AMI: acute myocardial infarction; AKI: acute kidney injury; PHEO: pheochromocytoma.

**Chart 2:** Differences in the diagnosis, prognosis and management of hypertensive urgency and emergency

Urgency	Emergency
Markedly high BP levelDBP > 120 mm Hg	Markedly high BP levelDBP > 120 mm Hg
Without acute and progressive TOD	With acute and progressive TOD
Oral drug combination	Parenteral medication
No risk of imminent death	Risk of imminent death
Early ambulatory follow-up care (7 days)	ICU admission

ICU: intensive care unit.

## Major epidemiological, pathophysiological and prognostic aspects

### Epidemiology

Hypertensive crisis accounts for 0.45-0.59% of all hospital emergency treatments, while HE accounts for 25% of all cases of HC, ischemic stroke and APE, which are the most frequent HEs.^4-6^

### Pathophysiology

Increased intravascular volume and PVR, or reduced production of endogenous vasodilators seem to precipitate greater vascular reactivity, resulting in HC.^7^ Self-regulation is compromised, particularly in the cerebral and renal vascular beds, resulting in local ischemia, which triggers a vicious circle of vasoconstriction, myointimal proliferation and target-organ ischemia.^8^

### Prognosis

Survival up to 5 years is significantly higher in individuals with HU than with HE.^4,9^ Absence of nocturnal dipping associates with higher risk for TOD and consequent endothelial dysfunction, a situation involved in acute BP elevation.^10^

## Complementary clinical and laboratory investigation

Clinical and laboratory investigation should properly assess BP and TOD. Initially, BP should be measured in both arms, preferably in a calm environment, and repeatedly until stabilization (minimum of 3 measurements). Data on the patient's usual BP should be rapidly collected, as well as information on situations that can raise it (anxiety, pain, salt), comorbidities, use of antihypertensive drugs (dosage and adherence) or drugs that can increase BP (anti-inflammatory drugs, corticoids, sympathomimetic drugs, alcohol). A systematic approach helps to check for the presence of acute and progressive TOD:

**Cardiovascular system:** chest, abdominal or back pain or discomfort; dyspnea, fatigue and cough. Assessment of HR, heart rhythm, pulse changes, gallop rhythm, cardiac and vascular murmurs, jugular venous distension, and pulmonary, abdominal and peripheral congestion. Exams requested based on clinical findings and availability: ECG, electrocardiographic monitoring, O2 saturation, chest X ray, echocardiogram, myocardial necrosis markers, blood cell count with platelets, LDH-C, CT angiography and MRI.

**Nervous system:** dizziness, headache, impaired vision, hearing or speech, consciousness or coma level, agitation, delirium or confusion, focal deficits, neck stiffness, convulsion. Exams: tomography, MRI and lumbar puncture.

**Renal and genitourinary system:** changes in urine volume, micturition frequency or urine aspect, hematuria, edema, dehydration, abdominal masses and murmurs. Exams: urinalysis, serum creatinine, serum urea, Na+, K+, Cl-, blood gas analysis.

**Retinal exam:** papilledema, hemorrhages, exudates, vascular changes, such as spasms, pathological arteriovenous crossings, arterial wall thickening and silver- or copper-wire aspect.

## General treatment of hypertensive crisis

The treatment of HU should begin after a period of clinical observation in a calm environment, which helps to rule out the cases of pseudocrisis (treated with only rest or use of painkillers or tranquilizers). Captopril, clonidine and BBs are oral antihypertensives used to gradually reduce BP in 24-48 hours. The use of drops of rapid-release nifedipine capsules to treat HU should be banned, because it is neither safe nor effective, and causes rapid and marked BP reductions, which can result in tissue ischemia. The use of nifedipine for preeclampsia is currently debatable.

The treatment of patients with HE is aimed at rapid BP reduction to prevent the progression of TODs. Patients should be admitted to the ICU, on IV antihypertensives and be carefully monitored to prevent hypotension. The general recommendations for BP reduction for HE are:^2^

- ↓ BP ≤ 25% in the 1^st^ hour;
- ↓ BP 160/100-110 mm Hg in 2-6 hours;
- BP 135/85 mm Hg in 24-48 hours.

However, HEs should be approached considering the impaired system or target organ. Thus, each type of HE (CV, cerebral, renal or other) should be previously characterized before beginning specific antihypertensive therapy.

## Hypertensive emergency in special situations

Chart 3 shows the medications used for HE.

**Chart 3:** Medications used via parenteral route to treat hypertensive emergencies

Medications	Administration route and dosage	Beginning	Duration	Indications	Adverse events and precautions
SNP (arterial and venous vasodilator, stimulates cGMP formation)	Continuous IV infusion0.25-10 mg/kg/min	Immediate	1-2 min	Most hypertensive emergencies	Cyanide poisoning, severe hypotension, nausea, vomiting. Careful in kidney and liver failure and high intracranial pressure. Protect from light
Nitroglycerin (arterial and venous vasodilator, nitric oxide donor)	Continuous IV infusion 5-15 mg/h	2-5 min	3-5 min	Coronary insufficiency, left ventricular failure with APE	Headache, reflex tachycardia, tachyphylaxis, flushing, metahemoglobinemia
Metoprolol (selective BB)	5 mg IV (repeat 10/10 min, if necessary up to 20 mg)	5-10 min	3-4 h	Coronary insufficiency, acute aortic dissection (in combination with SNP)	Bradycardia, advanced atrioventricular block, HF, bronchospasm
Esmolol (ultra-rapid selective BB)	Attack: 500µg/kgintermittent infusion 25-50µg/kg/min ↑ 25µg/kg/min every 10-20 min. Maximum 300µg/kg/min	1-2 min	1-20 min	Acute aortic dissection (in combination with SNP),severe postoperative hypertension	Nausea, vomiting, 1st-degree atrioventricular block, bronchospasm, hypotension
* Phentolamine (alpha-adrenergic blocker)	Continuous infusion: 1-5 mg Maximum 15 mg	1-2 min	3-5 min	Excess of catecholamines	Reflex tachycardia, flushing, dizziness, nausea, vomiting
* Trimethaphan (SNS and PSNS ganglionic blocker)	Continuous infusion:0.5-1.0 mg/min. ↑ 0.5 mg/ min up to maximum of 15 mg/min	1-5 min	10 min	Excess of catecholamines Acute aortic dissection	Tachyphylaxis
Hydralazine (direct vasodilator)	10-20 mg IV or10-40 mg IM 6/6 h	10-30 min	3-12 h	Eclampsia	Tachycardia, headache, vomiting. Worsening of angina and infarction. Careful in high intracranial pressure
* Diazoxide (vasodilator of arteriolar smooth muscle)	Infusion 10-15min1-3 mg/kg Maximum 150 mg	1-10 min	3-18 h	Hypertensive encephalopathy	Retention of sodium, water, hyperglycemia and hyperuricemia
* Fenoldopam (dopaminergic agonist)	Continuous infusion0.1-1.6µg/kg/min	5-10 min	10-15 min	AKI	Headache, nausea, flushing
* Nicardipine (CCB)	Continuous infusion5-15 mg/h	5-10 min	1-4 h	Stroke,hypertensive encephalopathy,left ventricular failure with APE	Reflex tachycardia, phlebitis, avoid in patients with HF or myocardial ischemia
* Labetalol (alpha/beta-adrenergic blocker)	Attack: 20-80 mg 10-10 min Continuous infusion 2 mg/min (maximum 300 mg/24h)	5-10 min	2-6 h	Stroke,acute aortic dissection (in combination with SNP)	Nausea, vomiting, atrioventricular block, bronchospasm, orthostatic hypotension
* Enalapril (ACEI)	Intermittent infusion1.25-5.0 mg 6/6h	15 min	4-6 h	Left ventricular failure with APE	Hypotension, kidney failure
Furosemide (loop DIU)	20-60 mg (repeat after 30 min)	2-5 min	30-90 min	Left ventricular failure with APE, hypervolemia	Hypopotassemia

* Not available in Brazil. SNP: sodium nitroprusside; cGMP: cyclic guanosine monophosphate; SNS: sympathetic nervous system; PSNS: parasympathetic nervous system; APE: acute pulmonary edema; AKI: acute kidney injury; HF: heart failure; ACEI: angiotensin-converting-enzyme inhibitor; DIU: diuretic.

### Stroke

Arterial hypertension is the major risk factor for stroke, especially hemorrhagic stroke. The diagnosis is based on complete neurological exam. To assess the severity of the condition, the National Institute of Health Stroke Scale (NIHSS) should be used. Brain CT and MRI allow defining the type of stroke and territory involved, and, usually, 85% of the strokes are ischemic, and 15%, hemorrhagic.^11^ For incipient infarctions, MRI is more sensitive than CT.

### Hemorrhagic stroke12

1. For patients with SBP between 150 and 220 mm Hg and with no treatment contraindication, acute SBP reduction to 140 mm Hg is safe and can be effective to improve the functional outcome. (GR: IIa; LE: B) (in 1 hour with IV infusion of antihypertensives and BP monitoring 5/5 min) (GR: I; LE: A).
2. For patients with SBP > 220 mm Hg, consider aggressive BP reduction with continuous IV infusion and frequent BP monitoring. (GR: IIb; LE: C).

### Ischemic stroke13

1. For patients with no indication for thrombolytic therapy and initial BP > 220/120 mm Hg, BP should not be reduced more than 15-20%, maintaining DBP as 100-110 mm Hg in the first 24 hours.
2. The ideal BP level to be attained is not known, but there is consensus that no antihypertensive treatment should be instituted during the initial care, except if SBP is > 220 mm Hg or DBP is > 120 mm Hg. (GR: I; LE: C).
3. Consider the possibility of using thrombolytics after BP control. For patients with indication for thrombolytic therapy and initial BP > 185/110 mm Hg, BP should be reduced to < 185/105 mm Hg for, at least, the first 24 hours after the thrombolytic agent. (GR: I; LE: B).

### Acute coronary syndromes

Coronary syndromes can be accompanied by BP elevation, because of a reflex of the ischemic myocardium. The increased PVR increases myocardial oxygen demand because of the increased left ventricular wall tension.

The IV nitrates reduce PVR, improve coronary perfusion and have an important systemic vasodilator effect, reducing preload and myocardial oxygen consumption. SNP is not indicated because of the coronary flow steal mechanism caused by generalized coronary vasodilation.^2,3^

### Unstable angina / non-ST elevation MI / ST elevation MI14,15

To treat AH, persistent ischemia and HF, IV nitroglycerin is indicated in the first 48 hours. Its use should not exclude other interventions that have proven to reduce mortality, such as BBs or ACEIs. Nitroglycerin is, however, contraindicated in the presence of recent use of phosphodiesterase inhibitors (previous 24 to 48 hours). (GR: I; LE: B).

The IV use of BBs is indicated for individuals with AH who have no signs of HF, clinical evidence of low cardiac output, increased risk for cardiogenic shock or other contraindications relating to beta blockade. (GR: IIa; LE: B).

### Acute pulmonary edema

Approximately one third of the patients admitted with APE and HE have preserved left ventricular function. Myocardial ischemia can also be involved in the pathophysiology of the APE associated with HE.^16,17^ The HE with APE findings should be controlled in an ICU setting, with parenteral medication, monitoring and gradual BP decrease.^18^

#### Acute aortic dissection

Acute aortic dissection should always be considered in patients with precordial pain and BP elevation. Progression of the dissection is related to the BP level and ventricular ejection velocity.^19^ Target SBP (120 mm Hg) should be achieved in 20 minutes. The isolated use of SNP is not ideal, because it increases HR and the aortic ejection velocity, and can worsen the dissection. Thus, SNP should be associated with a BB. In case of intolerance to SNP or contraindication to BBs, trimethaphan should be used.

#### Use of illicit substances

Illicit substances that raise BP, such as cocaine, crack, amphetamines and ecstasy, have sympathomimetic action.^20^ Crack and cocaine increase the risk for stroke and acute coronary insufficiency.^21^ In addition to increasing HR and BP, ecstasy have other effects, mainly serotoninergic syndrome, and can cause rhabdomyolysis and AKI.^22^ A complicator of those intoxications is the concomitant ingestion of high doses of caffeine, present in energetic beverages, nicotine and alcohol. Those intoxications have in common the high level of plasma noradrenaline.^23^ The treatment includes the use of BBs, alpha-blockers and CCBs.^24^

#### Rapidly progressive acute kidney injury

Acute and progressive renal function impairment is observed in patients admitted to hospital emergency units.^25^ Individuals with greater renal function impairment have important cardiac dysfunction and greater loss of renal function during episodes of marked BP elevation, which is accompanied by high in-hospital mortality rates.^26^ Rapidly progressive AKI is defined as a sudden renal function worsening in 48 hours, with specific classification criteria: RIFLE (*Risk, Injury, Failure, Loss, End-Stage Kidney Disease*) and AKIN (*The Acute Kidney Injury Network*).^27^ Treatment includes hydralazine, loop DIUs and BBs. In case of no result, SNP can be considered until dialysis is performed.

The management for preeclampsia and eclampsia is reported in Chapter 9.
